# Missed foot fractures in multiple trauma patients

**DOI:** 10.1186/s12891-019-2501-8

**Published:** 2019-03-25

**Authors:** Stefanie Fitschen-Oestern, Sebastian Lippross, Rolf Lefering, Lutz Besch, Tim Klüter, Elke Schenzer-Hoffmann, Andreas Seekamp, TraumaRegister DGU

**Affiliations:** 1 Department of Trauma Surgery, University Medical Center of Schleswig-Holstein, Campus Kiel, Arnold-Heller Straße 7, 24105 Kiel, Germany; 2 Institute for Research in Operative Medicine (IFOM), University Witten/Herdecke, Cologne, Germany; 3 Committee on Emergency Medicine, Intensive Care and Trauma Management, (Sektion NIS) of the German Trauma Society (DGU), Cologne, Germany

**Keywords:** Multiple trauma, Missed foot injuries, TraumaRegister DGU®, Primary survey, Secondary survey, Tertiary survey

## Abstract

**Background:**

Missed or underestimated injuries are one of the central problems in trauma care. Foot injuries can easily be missed because they lay beyond the regularly screened field of a trauma computer tomography scan (CT scan). During primary and secondary survey a careful examination of the extremities often becomes of secondary interest in the severely injured patient.

**Methods:**

Thirty-four thousand ninety-one multiple trauma patients of the TraumaRegister DGU® were evaluated from 2002 to 2014. We differentiated between patients with foot injuries, patients with missed foot injuries and patients without foot injuries. Included were ankle fractures, calcaneus fractures, talus fractures, metatarsal fractures, toe fractures, amputation, soft tissue injuries and/or ligamentous injuries.

**Results:**

Summarized evaluation of 34,091 trauma patients showed a share of 2532 patients with foot injuries. Time of diagnosis was documented in 2199 cases. 2055 patients had early diagnosed foot injuries and 144 patients had initially missed foot injuries. Missed foot injuries were especially found in patients with car accidents or fall from ≥3 m. Patients with higher Abbreviated Injury Scale (AIS) or lower Glasgow Coma Scale (GCS) were not significantly more affected by missed foot injuries. Missing foot injuries was also not caused by injury severity or higher age.

**Conclusions:**

Our data highlights the need of careful evaluation of the feet during primary and secondary survey particularly when a tibia or femur fracture is diagnosed. Special attention should be turned to patients after car accidents or fall from great height. Suicide victims also need major attention. Patients with early operations also need careful examination and tertiary survey is highly recommended.

## Background

Missed injuries and delayed diagnosis are essential reasons for limited outcome of multiple trauma patients. Foot injuries are often missed in trauma patients and are a source of long-term limitation [1]. Injuries below the knee generally come along with high risk for unemployment, long sick leave and decreased outcome [1]. Missed injuries in trauma patients are one of the main topics in trauma care and were evaluated several times before. Especially patients with head injuries, unconsciousness with a Glasgow Coma Scale of eight or lower and a high. Injury Severity Score (ISS) are predisposed to have missed injuries or delayed diagnosis [2]. Injuries are often missed during the primary and secondary surveys in trauma patients [3]. Careful examination in the initial stage after severe injury can especially improve outcome of multiple trauma patients with lower extremity injuries.

Depending on the localisation there is a wide spread distribution of missed injuries and delayed diagnosis incidence rates from 1.3 to 39% [2]. The integration of computed tomography (CT) has essentially improved the process of trauma care and accuracy of diagnostic procedures in the last decades [4, 5] but injuries of the foot are not routinely detected in the standard trauma scan protocol. Several studies evaluated different missed foot fractures in separate level 1 trauma centers [6–8]. Depending on the study design the percentage of patients with missed foot injuries differs from 12.2 to 44.7% [6–8]. Contrary to other studies we focused on the The TraumaRegister DGU®.

## METHeODS

### TraumaRegister DGU®

The TraumaRegister DGU® of the German Trauma Society (Deutsche Gesellschaft für Unfallchirurgie, DGU) was founded in 1993. The aim of this multi-centre database is a pseudonymised and standardised documentation of severely injured patients.

Data are collected prospectively in four consecutive time periods from the site of the accident until discharge from hospital: A) Pre-hospital phase, B) Emergency room and initial surgery, C) Intensive care unit (ICU) and D) Discharge. The documentation includes detailed information on demographics, injury pattern, comorbidities, pre- and in-hospital management, course on intensive care unit, relevant laboratory findings including data on transfusion and outcome of each individual. The inclusion criterion is admission to hospital via emergency room with subsequent ICU/Intensive Care Medicine (ICM) or reaching the hospital with vital signs and death before admission to ICU.

The infrastructure for documentation, data management, and data analysis is provided by AUC - Academy for Trauma Surgery (AUC - Akademie der Unfallchirurgie GmbH), a company affiliated to the German Trauma Society. The scientific leadership is provided by the Committee on Emergency Medicine, Intensive Care and Trauma Management (Sektion NIS) of the German Trauma Society. The participating hospitals submit their pseudonymised data into a central database via a web-based application. Scientific data analysis is approved according to a peer review procedure established by Sektion NIS.

The participating hospitals are primarily located in Germany (90%), but a rising number of hospitals of other countries contribute data as well (at the moment from Austria, Belgium, China, Finland, Luxembourg, Slovenia, Switzerland, The Netherlands, and the United Arab Emirates). Currently, approx. 25.000 cases from more than 600 hospitals are entered into the database per year. Participation in TraumaRegister DGU® is voluntary. For hospitals associated with. TraumaNetzwerk DGU® however, the entry of at least a basic data set is obligatory for reasons of quality assurance. The present study is in line with the publication guidelines of the TraumaRegister DGU® and registered as TR-DGU project ID 2014- 027. The Ethical Committee Kiel, Schleswig-Holstein examined and approved the study (D415/18).

### Patients

Thirty-four thousand ninety-one multiple trauma patients were evaluated from 2002 to 2014 and data were analysed. All data were taken from the TraumaRegister DGU®. Included were all patients between 1 and 100 years of age. Secondary transfers were not considered. We included all participating hospitals within Germany. An injury/injuries of the feet could be verified in 2532 cases (7.4%). Included were ankle fractures with/without soft tissue injuries and/or ligamentous injuries, calcaneus fractures, talus fractures, metatarsale fractures, toe fractures and amputation.

Missed injuries were defined as injuries, which were not diagnosed during primary and secondary survey. Diagnosis was made after admission to ICU. For the evaluation of missed/not missed injuries, we included only patients with information of time of diagnosis. Several of these patients had more than one missed injury. In this case all missed injuries were included, but the number of multiple trauma patients with one or more missed injuries was analyzed.

### Statistical analysis

Statistics were calculated using SPSS 22.0. (IBM, IBM Deutschland GmbH) and Graph Pad Prism 7 (Graphpad Software, Inc., USA). For descriptive analyses, results are presented as the mean ± standard deviation (SD). Differences in the ratios between groups were tested using the chi-squared test, and Student’s t-test was used for significance testing if a normal distribution was found, or the Mann–Whitney U test if a normal distribution was absent. The unpaired t-test with Welch’s correction was used for calculated mean values with different standard deviation. Odds ratio was calculated for different variables. Confidence interval was respectively declared. The data are for continuous measurements and as totals (percentage) for categorical variables. Statistical significance was defined as *p* < 0.001. However, due to the large sample size very small *p* values result, thus *p*-values should be interpreted cautiously. Besides statistical significance, the clinical relevance of the observed differences always needs to be considered.

## Results

### Patients with foot injuries

**Fig. 1:**
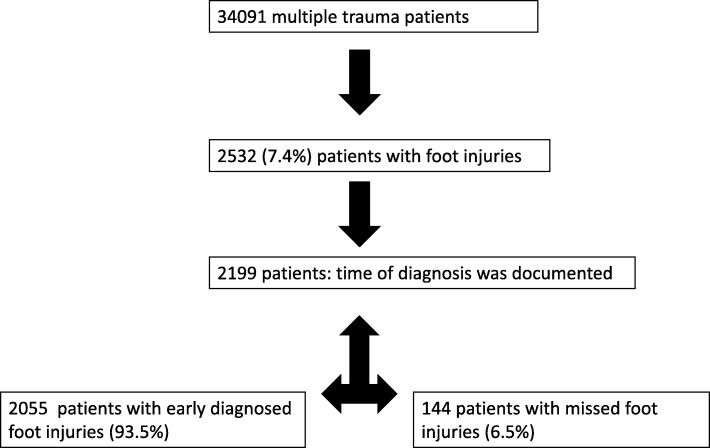
Incidence of multiple trauma patients with early diagnosed foot injuries and missed foot injuries

**Fig. 2:**
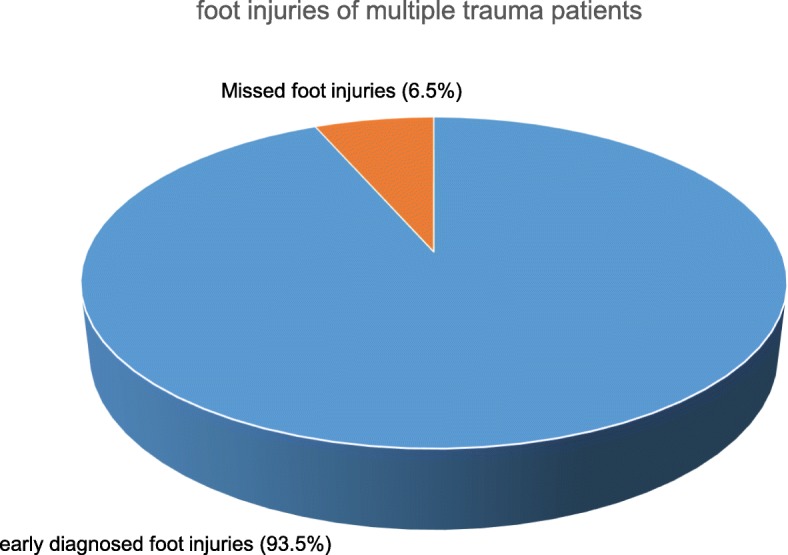
Share in early determined foot injuries and missed foot injuries

Thirty-four thousand ninety-one patients were evaluated with regard to foot injuries or no foot injuries (Figs. 1 and 2). Foot injuries were documented in 2532 cases (7.4%). Two thousand two hundred forty-seven patients (6.6%) sustained a foot fracture and 285 patients (0.8%) sustained a ligamental injury.

Table 1 shows basic parameters of multiple trauma patients with foot injuries and without foot injuries. Eight hundred three female patients (31.9%) and 1717 male patients (68.1%) suffered from foot injuries. Proportional gender distribution was similar in the group with versus without hand injuries.

**Table 1:** Basic characteristics, injury severity, early treatment and outcome of multiple trauma patients with and without foot injuries. The small *p* values are based on the large sample size and interpretation should implicate the clinical importance of observed difference

	Patient without foot injuries	Patients with foot injuries	Total
n	31,559	2532	34,091
gender female	8473 (27%)	803 (31.9%)	9276
male	22,918 (73%)	1717 (68.1%)	24,635
age 1 to 17 years	1972 (6.3%)	102 (4.1%)	2074
18 to 59 years	18,662 (59.5%)	1842 (73.2%)	20,504
60 to 69 years	7252 (23.1%)	439 (17.4%)	7691
> 70 years	3505 (11.2%)	134 (5.3%)	3639
cause of accident car	9231(30.5%)	989 (40.1%)	10,220
motorcycle	4649 (15.4%)	352 (14.3%)	5001
bicycle	2510 (8.3%)	45 (1.8%)	2555
pedestrian	2816 (9.3%)	119 (4.8%)	2935
fall > 3 m	5863 (19.4%)	827 (33.5%)	6690
fall < 3 m	3047 (10.1%)	49 (2%)	3096
others	2157 (7.1%)	85 (3.4%)	2242
ISS 16–24	12,945 (41%)	1145 (45.2%)	14,090
ISS 25–34	10,678 (33.8%)	804 (31.8%)	11,482
ISS 35–49	5202 (16.5%)	394 (15.6%)	5596
ISS 50–75	2734 (8.6%)	189 (7.5%)	2923
GCS > 8	16,940 (67%)	1580 (78.8%)	18,520
GCS ≤ 8	8329 (33%)	423 (21.1%)	8752
AIS head < 3	14,201 (45%)	1682 (66.4%)	15,883
≥ 3	17,358 (55%)	850 (33.6%)	18,208
AIS thorax< 3	10,467 (33.2%)	872 (34.4%)	11,339
≥ 3	21,092 (66.8%)	1660 (65.6%)	22,752
AIS abdomen < 3	24,664 (78.2%)	1747 (69%)	26,411
≥ 3	6895 (21.9%)	785 (31%)	7680
GOS dead	5366 (17.9%)	235 (9.9%)	5601
persisted vegetative state	767 (2.6%)	31 (1.3%)	798
severely handicapped	3573 (11.9%)	344 (14.4%)	3917
slightly handicapped	7677 (25.6%)	859 (36.1%)	8536
well recovered	12,640 (42.1%)	912 (38.3%)	13,552
discharge from hospital to home	11,077 (35.2%)	775 (30.7%)	11,852
to rehabilitation clinic	10,601 (33.7%)	973 (38.5%)	11,574
to another hospital	3585 (11.4%)	439 (17.4%)	4024
others	802 (2.6%)	102 (4.0%)	904
death	5366 (17.1%)	235 (9.3%)	5601

**Table 2:** Clinical characteristics, injury severity, early treatment and outcome of multiple trauma patients with early diagnosed and missed foot injuries. *P* values are shown in the table

	Patients with early diagnosed foot injuries	Patients with missed foot injuries	Total	p
n	2055	144	2199	
gender female	545 (31.7%)	39 (31.2%)	584	0,9932
male	1174 (68.3%)	86 (68.8%)	1260	
age 1 to 17 years	72 (4.2%)	6 (4.8%)	78	0,9809
18 to 59 years	1261 (73.4%)	87 (69.6%)	1348	
60 to 69 years	286 (16.6%)	25 (20.0%)	311	
> 70 years	99 (5.8%)	7 (5.6%)	106	
cause of accident car	691 (40.9%)	54 (44.3%)	745	0,835
motorcycle	240 (14.2%)	16 (13.1%)	256	
bicycle	28 (1.7%)	3 (2.5%)	31	
pedestrian	80 (4.7%)	8 (6.6%)	88	
fall > 3 m	567 (33.5%)	31 (25.4%)	598	
fall < 3 m	30 (1.8%)	5 (4.1%)	35	
others	55 (3.3%)	5 (4.1%)	60	
GCS > 8	1292 (79.1%)	104 (84.6%)	1396	0,3488
GCS ≤ 8	342 (20.9%)	19 (15.4%)	361	
AIS head < 3	1147 (66.5%)	76 (60.8%)	1223	0,4355
≥ 3	579 (33.5%)	49 (39.2%)	628	
AIS thorax< 3	581 (33.7%)	45 (36%)	626	0,8673
≥ 3	1145 (66.3%)	80 (64.0%)	1225	
AIS abdomen < 3	1210 (70.1%)	85 (68%)	1295	0,8844
≥ 3	516 (29.9%)	40 (32%)	556	
GOS dead	172 (10.5%)	10 (8.4%)	182	0,9735
persisted vegetative state	21 (1.3%)	1 (0.8%)	22	
severely handicapped	234 (14.3%)	13 (10.9%)	247	
slightly handicapped	590 (36%)	44 (37%)	634	
well recovered	624 (38%)	51 (42.9%)	675	
discharge from hospital to home	538 (31.2%)	46 (37.1%)	584	0,6108
to rehabilitation clinic	655 (38%)	44 (35.5%)	699	
to another hospital	289 (16.8%)	15 (12.1%)	304	
others	69 (4.0%)	9 (7.3%)	78	
death	172 (10.0%)	10 (8.1%)	182	

Most patients included in the study were 18–59 years of age 1842 patients with foot injuries and 18,662 without foot injuries were at the age 18–59 years**.** Proportionally patients with foot injuries (73.2%) were more often at the age 18–59 years compared to patients without foot injuries (59.4%).

Patients with foot injuries (2168 patients (87.9%)) were proportionally more involved in high energy trauma compared to patients without foot injuries (19,743 patients (65.2%)) (*p* < 0.001).

The most common cause of injury in patients with or without foot injuries were car accidents (989 patients with foot injuries (40.1%), 9231 patients without foot injuries (30.5%)) or fall > 3 m (827 patients with foot injuries (33.5%), 5863 patients without foot injuries (19.4%)) (*p* < 0.001).

**Fig. 3:**
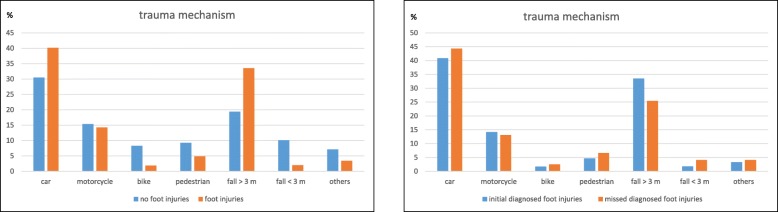
Distribution of the cause of the accident for multiple trauma patients with and without foot injuries

Five hundred eleven patients with foot injuries (20.5%) and 1724 patients without foot injuries (5.6%) tried to commit suicide (*p* < 0.001) (Table 1 and Fig. 3).

We determined if severity of multiple trauma correlates with the presence of foot injuries. We investigated parameters of ISS, GCS and AIS for head, chest and abdomen. Unconsciousness was defined by an initial GCS ≤ 8 [9].

Distribution of ISS was similar in trauma patients with foot injuries and without foot injuries.

Multiple trauma patients with foot injuries did not tend to have lower scores of GCS or higher AIS for head injuries. Four hundred twenty-three patients with foot injuries (21.1%) and 8329 patients without foot injuries (33%) were admitted to hospital with a GCS ≤ 8 (*p* < 0.001). Distribution of AIS chest was similar in both groups, whereas proportional trauma patients with foot injuries were more affected by abdomen trauma (Table 1).

We focused on combination of foot injuries with tibia fracture, fibula fracture or femur fracture. Nine hundred thirty patients with foot injuries (36.7%) and 4174 patients without foot injuries (13.3%) had also a tibia fracture (*p* < 0.001). Nine hundred forty-nine patients with foot injuries (37.5%) and 5459 patients (17.3%) without foot injuries had a femur fracture (*p* < 0.001). A fibula fracture was documented in 471 cases (18.6%) with foot injuries and in 1673 cases (5.3%) without foot injuries (*p* < 0.001).

More patients died in the group without foot injuries than in the foot-injury group (5366 patients (17%) versus 235 patients with foot injuries (9.3%)) (*p* < 0.001).

Regarding the percentage distribution, more patients with foot injuries (859 patients (36.1%)) than patients without foot injuries (7677 patients (25.6%) were discharged from the hospital as moderate disabled (*p* < 0.001).

The majority of patients with or without foot injuries were discharged home (11,077 patients without foot injuries (35.2%), 775 patients with foot injuries (30.7%)) or to a rehabilitation clinic (*p* < 0.001).

### Patients with missed foot injuries

Time of diagnosis was only documented in 2199 cases of patients with foot injuries (6.5%). Two thousand fifty-five patients with foot injuries (93.5%) were identified in the emergency room and 144 patients with injuries (6.5%) were first observed at the ICU (Table 2,  Figs. 1 and 2).

Our analysis showed 115 amputations, 174 ankle fractures, 1063 calcaneus fractures, 944 metatarsal fractures, 518 talus fractures and 331 toe fractures, which were diagnosed at the emergency room. Eight ankle fractures, 63 calcaneus fractures, 65 metatarsal fractures, 34 talus fractures and 31 toe fractures were diagnosed at ICU. Data about further medical care were found for 165 late diagnosed and 2367 early diagnosed foot injuries. Operative therapy was documented for 93 late diagnosed (56.4%) and 1404 early diagnosed foot injuries (59.3%). Difference was not significant.

The average age of patients with early diagnosed foot injuries were 41.3 ± 17.9 years whereas patients with missed foot injuries had an average age of 40.8 ± 18.7 years old. Difference was not significant (Table 2).

The majority of missed foot injuries also occurred after car accidents (54 cases (44.3%)) or fall > 3 m (31 cases (25.4%)) (Table 2). Proportional distribution for comitted suicide was similar in both groups (19 patients with missed foot injuries (15.4%), 354 patients with early diagnosed foot injuries (20.8%)).

Average ISS of patients with early diagnosed foot injuries was 28.8 ± 11.4 and average ISS of patients with missed injuries was 28.5 ± 11.7. Difference was not significant.

Decision of transfer to ICU or operative treatment was made in the emergency room. After emergency room management, 425 patients (26.9%) with early diagnosed foot injuries and 38 patients with missed injuries (33%) were transfere to ICU. The majority of patients was operated within the first 24 h (early operation) (1054 patients with early diagnosed foot injuries (66.6%) versus 64 patients (55.7%) with missed foot injuries). Interruption of emergency room management and an emergency operation was necessary for 70 patients with early diagnosed foot injuries (4.4%). Difference was not significant.

Complete hospital stay of patients with foot injuries documented at the emergency room took 39.9 ± 29.6 days (median 32) (15.1 ± 15.7 days at the ICU (median 10.5)). Patients with a missed foot injuries stayed 28.5 ± 25.3 days at hospital (median 22) (12.8 ± 13.9 days at the ICU (median 8.0)) (*p* < 0.001).

Six hundred ninety-six patients with early diagnosed foot injuries (40.5%) and 40 patients with missed foot injuries (32%) received blood supply. Difference was not significant.

Lack of diagnostic or early termination of trauma management can be a reason for missed injuries. Multislice-body-CT was documented in the majority of trauma cases with early diagnosed and missed foot injuries. 42 patients (33.9%) with missed foot injuries had no CT scan whereas 373 patients with early diagnosed foot injuries (21.7%) did not receive a CT scan (p < 0.001).

In both groups, the majority of patients left the hospital well recovered (624 (38%) patients with early diagnosed foot injuries, 51 (42.9%) patients with missed foot injuries) or slightly handicapped (590 (36%) patients with early diagnosed foot injuries, 44 (37%) patients with missed foot injuries). Difference was not significant.

Additionally we evaluated where to patients were discharged and if missed injuries might cause a delayed discharge home. 538 (31.2%) patients with early diagnosed foot injuries and 46 (37.1%) with late diagnosed foot injuries went home. 655 (38%) patients with early diagnosed foot injuries and 44 (35.5%) with late diagnosed foot injuries were transferred to a rehabilitation unit. Difference was not significant.

We also calculated the odds ratio for missed foot injuries related to different parameters. Positive odds ratio could be detected for suicide OR 2.7 [2.3–3.1] and for additional injuries like tibia fracture, OR 2.5 [2.3–2.8], femur fracture, OR 1.7 [1.5–1.8] and fibula fracture, OR 2.6 [2.3–3]. Age of 18 to 54 showed a positive OR of 1.5 [1.2–1.8] and a positive odds ratio, OR 1.4 [1.3–1.6], could be detected for all patients with early operations.

## Discussion

Missed injuries and delayed diagnoses are still serious problems in the treatment of multiple trauma patients. For minimizing its occurrence, it is essential to understand the etiology of missed injuries.

We focused on foot injuries, while these injuries are not assessed by standard Polytrauma-CTscan protocols but represent essential reasons for limited outcome of multiple trauma patients. Our study was conducted to identify the incidence, contributing factors and clinical outcomes of patients with foot injuries, especially when they were missed.

The incidence of missed foot injuries in multiple trauma patients shows high variation according to type of injury, country and time interval. Houshian et al. showed a proportion of 12.8% for missed foot and ankle fractures [10], while Guly et al. showed a proportion of 25.8% [11]. Due to improving standards and procedures in the emergency department part of missed injuries could already be reduced over the last decades.

We evaluated 34,091 trauma patients from 2002 to 2014, foot injuries were documented in 2532 cases (7.4%). Time point of diagnosis was not documented in all cases and not all foot injuries were documented in the TraumaRegister DGU®. This seems to be a weak point of all data bases in general. A delayed diagnosis on ICU was documented in 144 cases. Injuries of the foot and ankle region documented in the TraumaRegister DGU® have been evaluated before [12] but missed foot injuries are in focus for the first time.

Compared to a younger population, the treatment of older trauma victims is generally known to be associated with a higher rate of complications, higher mortality and morbidity [13]. Correlation of missed injuries and patient gender and age has also been shown before [14]. In our evaluation, most foot injuries occur at the age between 18 and 59 years while most missed injuries were also documented in this time period. We did not find a correlation between higher age and missed foot injuries.

Male patients were more affected by foot injuries compared to female patients while male patients show also a higher portion in multiple trauma in general.

### Mechanism of injury

The mechanism of injury is of vital importance and may give valuable clues towards diagnosis of injuries. Most foot injuries occur after car accidents or fall from great height. In view of cause of injury, we did not find a significant difference between patients with early diagnosed and missed injuries. Patients with car accidents hold the majority of foot injuries [6]. Although the overall car passenger safety has improved over the last decades the relative incidence of foot injuries has increased [15]. Morgan et al. analysed resulting trauma after car crash to various body regions to reveal that the greatest risk of injury is to foot and ankle, leg, pelvis and chest [16].

Fall from height is another common mechanism of foot injury. Atanasijevic et al. supports the hypothesis that the frequency and extent of the injuries are related to the fall height [17]. Respectively fall of a height of ≥3 m hold higher incidence for foot injuries [17]. Fall of great height is generally accompanied by multiple lifethreatening injuries so that careful examination of the feet is often secondary.

Especially suicide cases hold great prevalence of foot injuries. Fall from a height has been described as the most frequent mechanism of self-inflicted trauma [18]. Suicide victims generally jump feet first. Several studies showed that lower extremities are the most frequent areas involved in those patients [19]. Rissen et al. indicated that suicides were linked to greater heights than accidents [20].

### Severity of injury

Patients with missed injuries tend to be more severely injured with initial neurologic compromise [21]. A substantial correlation between higher severity of trauma (AIS) and/or a decreased consciousness (GCS) and a higher rate of delayed diagnosed foot injuries could be provided [22, 2]. These patients are often uncooperative or unresponsive and are unable to reflect valuable detail of the event or history data.

We could not verify any significant correlation between higher AIS or lower GCS and missed foot injuries, which seems to be confusing initially. Brooks et al. showed in a recent study that there are no differences in score systems between patients with missed injuries and patients without missed injuries [23]. This might be caused by the fact, that especially trauma patients with life-threatening injuries and high ISS are examined more carefully. The absence of a CTscan plays also a role in missing foot injuries. Predisposing factors for missing injuries might also have changed over the last decades.

Missed foot injuries might be a result of a priorisation that takes place during the initial assessment at the emergency department. The American College of Surgeons developed the Advanced Trauma Life Support (ATLS) course to evaluate the trauma patient with a systematic examination that utilizes primary and secondary surveys. Complete injury identification during resuscitation including primary and secondary survey is not always granted [24, 25]. A tertiary survey within 24 h has reduced the risk of missed injuries generally [26] and has become more and more common in the last years. It is defined as a patient evaluation that identifies and catalogues all injuries after the initial resuscitation and operative intervention [27]. The time point of the tertiary survey is institution specific but it always includes the repetition of the primary and secondary surveys and a review of radiographic studies with an attending radiologist [28]. Implementation of tertiary survey has essentially decreased missed injuries especially in the severely injured patients [24].

Even so tertiary survey is not always guaranteed and all surveys are not documented in the TraumaRegister DGU®. Probability of implementation is however higher in the severely injured patients with long-term ventilation.

Majority of patients with missed foot injuries had an early operation which might have caused an absence of reevaluation or delayed teriary survey. Besides inaccurate or not repeated clinical examination, missing or inadequate x-rays seem to be another major problem [29]. Concerning these facts a CTscan should be performed after careful examination if foot injuries are suspected. Patients with tibia or femur fractures have a higher prevalence of foot injuries. This fact might support the conclusion that missed extremity injuries are more often found in patients with multiple injured extremities. Ward et al. pointed out that hastily applied emergency splints might obscure a less apparent extremity injury as potential etiology of avoidable type of missed injury [30]. Unstable long bone fractures and swollen and painful soft tissue might also distract from further injuries during primary and secondary surveys. Further operative care of tiba or femur fractures is generally placed as early operation.

Majority of evaluated patients with missed foot injuries was discharged home or to a rehabilitation clinic. Nevertheless this fact does not predict which severely injuredpatients have good chances to recover completely and which not. Especially„bagatelle lesions “of the lower extremities were announced to limit activities of daily life [31]. Concerning body functions majority of patients declare a loss of function inactivities of daily life and working ability [31]. GOS was determined before patientswere transfered for further rehabilitation. Data for GOS were genereally evaluated before patients left the hospital. Patients with foot injuries showed a high percental share inslightly handicapped patients. Slightly handicapped is defined as living without any adjuvants and working in special facilities is possible which represents an extensive limitation in daily life.

In view of detailed longterm-outcome analysis further evaluations will be necessary.

### Limitation of the study

The TraumaRegister DGU® differentiates between injuries identified in the emergency room and injuries first observed at the ICU. Injuries which are diagnosed after discharge from hospital are not documented.

We focused especially on patients with missed foot injuries. The TraumaRegister DGU® did not provide information about the number of missed injuries of every single patient.

Data bases of multiple trauma patients provide AIS codes. There is no exact code for each injury type of the foot. Information about exact diagnosis of ligamentous/tendon/muscle injuries or luxation is not provided by the TraumaRegister DGU®.

## Conclusion

Summarized evaluation of 34,091 trauma patients and 2532 patients with foot injuries in a time period from 2002 to 2014 showed the following main findings:

-Missed foot injuries were especially found in patients with car accidents or fall from great height.

-Suicide patients were significantly affected by foot injuries.

-Patients with a higher AIS score or lower GCS were not affected more often from missed foot injuries compared to other patients.

-ISS does not play an essential role in missing foot injuries.

-Patients with foot injuries had significantly more often tibia or femur fractures.

Despite improvement of polytrauma management correct and careful primary, secondary and tertiary survey is essential. A tertiary survey within 24 h is indispensable. Early and accurate diagnosis of foot injuries may improve long-termoutcomes.

## Abbreviations

AIS: Abbreviated Injury Scale
ATLS: Advanced Trauma Life Support
AUC: Academy for Trauma Surgery
CTscan: computer tomography scan
DGU: German Trauma Society
GCS: Glasgow Coma Scale
GOS: Glasgow Outcome Scale
ICM: Intensive Care Medicine
ICU: Intensive Care Unit
IFOM: Institute for Research in Operative Medicine
ISS: Injury Severity Scale
Sektion NIS: Care and Trauma Management
TraumaRegister DGU®: Trauma Register der Deutschen Gesellschaft für Unfallchirurgie
Committee on Emergency Medicine: Intensive

## References

[1] Burns P, Highlander P, Shinabarger AB (2014). Management in high-risk patients. Clin Podiatr Med Surg.

[2] Pfeifer R, Pape HC (2008). Missed injuries in trauma patients: a literature review. Patient Saf Surg.

[3] Tammelin E, Handolin L, Söderlund T. Missed Injuries in Polytrauma Patients after Trauma Tertiary Survey in Trauma Intensive Care Unit. Scand J Surg. 2016. 10.1177/1457496915626837 26929292

[4] Sampson MA, Colquhoun KBM, Hennessy NLM (2006). Computed Tomography whole Body Imaging in Multi-Trauma: 7 Years Experience. Clin Radiol.

[5] Pereira SJ, O'Brien DP, Luchette FA, Choe KA, Lim E, Davis K, Hurst JM, Johannigman JA, Frame SB. Dynamic Helical Computed Tomography Scan Accurately Detects Hemorrhage in Patients with Pelvic Fracture. Surgery 2000; 128:678–685. 10.1067/msy.2000.108219 11015102

[6] Ahrberg AB, Leimcke B, Tiemann AH, Josten C, Fakler JK (2014). Missed foot fractures in polytrauma patients: a retrospective cohort study. Patient Saf Surg.

[7] Vles WJ, Veen EJ, Roukema JA (2003). Consequences of delayed diagnoses in trauma patients. a prospective study. J Am Coll Surg.

[8] Kalemoglu M, Demirbas S, Akin ML, Yildirim I, Kurt Y, Uluutku H, Yildiz M (2006). Missed Injuries in Military Patients with Major Trauma: Original Study. Military Medicine.

[9] Grote S, Böcker W, Mutschler W, Bouillon B, Lefering R (2011). Diagnostic value of the Glasgow Coma Scale for traumatic brain injury in 18,002 patients with severe multiple injuries. J Neurotrauma.

[10] Houshian S, Larsern MS, Holm C (2002). Missed injuries in a level I trauma center. J Trauma.

[11] Guly HR (2001). Diagnostic errors in an accident and emergency department. Emerg Med J.

[12] Probst C, Richter M, Lefering R, Frink M, Gaulke R, Krettek C, Hildebrand F.Incidence and significance of injuries to the foot and ankle in polytrauma patients--an analysis of the Trauma Registry of DGU. Injury 2010 Feb;41(2):210–215. 10.1016/j.injury.2009.10.009 19889412

[13] Aldrian S, Nau T, Koenig F, Vécsei V (2005). Geriatric polytrauma. Wien Klin. Wochenschr.

[14] Matuszak SA, Baker EA, Stewart CM, Fortin PT. Missed peritalar injuries: an analysis of factors in cases of known delayed diagnosis and methods for improving identification. Foot Ankle Spec 2014;7(5):363–371. 10.1177/1938640014537302 25037956

[15] Richter M, Thermann H, Wippermann B, Otte D, Schratt HE, Tscherne H (2001). Foot fractures in restrained front seat car occupants: a long-term study over twenty-three years. J Orthop Trauma.

[16] Morgan RM, Cui C, Digges KH, Cao L, Kan CD (2012). Impact and injury patterns in between-rails frontal crashes of vehicles with good ratings for frontal crash protection. Ann Adv Automot Med.

[17] Atanasijevic TC, Savic SN, Nikolic SD, Djoki VM. Frequency and severity of injuries in correlation with the height of fall. J Forensic Sci 200550(3):608–612. 15932094

[18] David JS, Gelas-Dore B, Inaba K (2007). Are patients with self-inflicted injuries more likely to die?. J Trauma.

[19] Rocos B, Chesser TJ (2016). Injuries in jumpers - are there any patterns?. World J. Orthop.

[20] Rissen D, Bonsch A, Schneider B, Bauer G. Risk of dying after a free fall from height. Forensic Sci Int 1996; 78: 187–191. 10.1016/0379-0738(95)01885-9 8635762

[21] Buduhan G, McRitchie DI (2000). Missed injuries in patients with multiple trauma. J.Trauma.

[22] Rizoli SB, Boulanger BR, McLellan BA, Sharkey PW. Injuries missed during initial assessment of blunt trauma patients. Accid Anal Prev 1994; 26:681–686. doi: 10. 10.1016/0001-4575(94)90030-2 7999213

[23] Brooks A, Holroyd B, Riley B (2004). Missed injury in major trauma patients. Injury.

[24] Biffl WL, Harrington DT, Cioffi WG (2003). Implementation of a tertiary trauma survey decreases missed injuries. J Trauma.

[25] Vles WJ, Veen EJ, Roukema JA, Meeuwis JD, Leenen LPH (2003). Consequences of Delaed Diagnoses in Trauma Patients A Prospective Study. J Am Coll Surg.

[26] Keijzers GB, Campbell D, Hooper J, Bost N, Crilly J, Steele MC, Del Mar C, Geeraedts LM Jr. A prospective evaluation of missed injuries in trauma patients before and after formalising the trauma tertiary survey. World J Surg 2014 Jan;38(1):222–232. 10.1007/s00268-013-2226-z PMC3889299 24081533

[27] Grossman MD, Born C (2000). Tertiary survey of the trauma patient in the intensive care unit. Surgical Clinics of North America.

[28] Janjua KJ, Sugrue M, Deane SA (1998). Prospective evaluation of early missed injuries and the role of tertiary trauma survey. The Journal of Trauma, Injury, Infection, and Critical Care.

[29] Wei CJ, Tsai WC, Tiu CM, Wu HT, Chiou HJ, Chang CY. Systematic analysis of missed extremity fractures in emergency radiology. Acta Radiol 2006 Sep;47(7):710-771. 10.1080/02841850600806340 16950710

[30] Ward WG, Nunley JA (1991). Occult orthopaedic trauma in the multiply injured patient. J Orthop Trauma.

[31] von Rüden C, Woltmann A, Röse M, Wurm S, Rüger M, Hierholzer C, Bühren V (2013). Outcome after severe multiple trauma: a retrospective analysis. J Trauma Manag Outcomes.

